# 

**Published:** 1843-06

**Authors:** 


### WESTERN JOURNAL

OF

### MEDICINE AND SURGERY.

### EDITORS.

---

DANIEL DRAKE, M. D.,

PROFESSOR OF PATHOLOGICAL ANATOMY AND CLINICAL MEDICINE
IN THE MEDICAL INSTITUTE OF LOUISVILLE:

LUNSFORD P. YANDELL, M. D.,

PROFESSOR OF CHEMISTRY AND PHARMACY IN THE SAME:

THOMAS W. COLESCOTT, M. D.

### INDEX TO VOL. VII.

A

Abnormal nutrition, 376
Abolition of a rule in medical graduation, 68
Abscess in the walls of the uterus, 307
Air, introduction of, into the veins, 460
Alabama, University of, 473
Alabama, first surgery of, 475
Amputation at the shoulder-joint, 405
Amaurosis, asthenic, cured by convex spectacles, 457
Ammonia in scirrhus of the stomach, 463
Ammonia in hemicrania, 227
Analysis of the menstrual fluid, 59
Anatomical injections, new process for, 295
Aneurism, false, variety of, 283
Animalculæ, revivification of, 59
Animals and man, sleep of, 233
Anonymous pamphleteers, 319
Antidote for corrosive sublimate, 230
Arkansas, medical topography of, 321
Army medical board, 476
Arsenic, effects of, on sheep, 462
Arteries, fatty degeneration of, 460
Arteries, structure of, 458
Artificial anus, 316
Assault on the temperance reform, 398
Asphyxia producing pneumonia, 422

B

Ballard's case of pericarditis, 329
Bars on steamboats, 238
Barton, Dr., removal of, to cuba, 313
Bayless on pneumonia, 422
Bay of Pensacola, 393
Bed and banks of the Mississippi, 237
Bed-sores, nitrate of silver in, 386
Bites of snakes, 65
Blind, school for, 77
Blood, researches on, 466
Botanical doctors in the south, 470
Buboes, primary, 456
Bursa, patellar, operation for, 229

C.

Calculus of the bladder simulated, 467
Calomel and botanical doctors, 470
Cæsarean operation on a dwarf, 340
Caries of the teeth, cause of, 297
Cases and observations, miscellaneous, 332
Cases of tardy fecundity, 408
Cathartics in retention of the placenta, 345
Causes and treatment of goitre, 456
Chancres, treated by sulphate of copper and cyanuret of mercury, 385
Charity Hospital, New Orleans, report of, 159
Chigoe or Gigger, 301
Children, new-born, signs of maturity in, 61
Churchill on diseases of females, 281
Climate, &c., of Central Arkansas, 321
Climate of New Orleans, 240
Climate of the United States, by Forry, 142
Colic from lead, prevention of, 297
Colony of lunatics, 465
Congestive fever, 412, 469
Congenital small-pox, 391
Consequences of introduction of glass into the body, 286
Consumption in man and animals, 381
Contusions of muscles, 302
Convention, Medical, of Ohio, 315, 429
Communication of pulmonary veins and air vesicles, 371
Cornea, peculiar affection of in nurses, 61
Corpora lutea, 60
Correspondents, notice to, 79
Corrosive sublimate in erysipelas, 72
Corrosive sublimate, new antidote for, 230

Crooked nose cured by subcutaneous division of cartilages, 231
Cubebs, cases of poisoning from, 357

D

Deafness cured by the endermic use of morphia, 54
Death of Tiger-Tail, 315
Death from diseased ear, 288
Deposits of tubercular matter in the bronchial glands, 380
Diagrams, surgical, 158
Digestion, researches on, 365
Dipping, 472
Discovery of the true cause of Milksickness, 73
Diseases, climate, &c., of Little Rock, 321
Diseases of females, by Churchill, 281
Diseases and temperature of 1842, 64
Diseases of the throat in public speakers, 66
Diseases, western, 236
Diseases of the eye, by Lawrence, 427
Doctors, calomel doctors, and botanical doctors. 470
Dowler on post-mortem researches, 241
Dropsy of the os uteri, 392
Drummond's Ricord on venereal diseases, 426
Dry feet, 461

E

Effects of arsenic upon sheep, 462
Electro-magnetism, 472
Electro-magnetism in a case of poisoning, 357
Electro-puncture. 467
Encephalo-spinal meningitis, 290
Endermic use of morphia in deafness, 54
Engorgement of the uterus, 225
Enlarged patellar bursa, operation for, 229
Existence of lymphatics in pseudo-membranes, 384
Explosions of steamboats, 240
Extraordinary case of pericarditis, 329

F

Falconer's Cæsarean operation, 340
False aneurism, variety of, 283
Fatty degeneration of the arteries, 460
Fecundity, tardy, cases of, 408
Feet, how to keep dry, 461
Females, Churchill on diseases of, 281
Fever, congestive, 412, 469
Fluid, menstrual, analysis of, 59
Forceps, galvanic, 292
Forry on the climate of the United States, 142
Forts at Pensacola, 394

G

Galvanic forceps, 292
Geological survey of Alabama, 473
Gigger or Chigoe, 301
Glanders in man, cure of, 388
Glass in the body, consequences of, 286
Goitre, causes and treatment of, 456
Goulding on the medical topography of Central Arkansas, 321
Graduation, medical, abolition of a rule in, 68
Gross on the nature and treatment of wounds of the intestines, 1, 81, 161

H

Harmony in the medical profession at the south, 473
Havana Medical Repertory, 476
Hay's Lawrence on the eye, 427
Hemicrania, muriate of ammonia in, 227
Hemp, Indian, 386
Hemorrhagic diathesis, treatment of, 53
Hernando de Soto, 475
Hospital at Pensacola, 394
Hospital liquors, waste of, 398
Hospital reports, St. Louis, 401, 476
Hydrophobia, idiopathic, 227
Hydrocele, improved treatment of, 464

I

Idiopathic hydrophobia, 227
Indian hemp, 386
India-rubber life-preservers, 239
Infants, nursery treatment of, 293
Injections, anatomical, new process for, 295
Intestines, memoir on wounds of, 1, 81, 161
Introduction of air into the veins, 460
Invalids, winter resort for, at Mammoth Cave, 78
Invalids, winter and spring visits of, to New Orleans, 314
Invention, rage for, 468
Iodide of potassium in atonic ulcers, 299
Iron, protosulphuret of, new antidote for corrosive sublimate, 230
Itch, treatment of, at Berlin, 231

L

Lancet, New York, 320
Latent insanity, 311
Lawrence on the eye, 427
Lawrence on ruptures, 428
Life-preservers, 239
Lithotomy on a man seventy-eight years of age, 78
Litton and Schnederman, on a new double salt, 154

Little Rock, Goulding on climate and diseases of, 321
Local treatment of chancres by sulphate of copper and cyanuret of mercury, 385
Louisiana, Medical College of, 314
Louisville Medical Institute, 316
Lunatics, colony of, 465
Luxation of the patella on its axis, 375
Lymphatics in false membranes, 384

M

Mammoth Cave, winter resort for invalids, 78
Materia Medica, Paine's therapeutical arrangement of, 237
Maturity in new-born children, signs of, 61
Medical College of Louisiana, 314
Medical Convention of Ohio, 315
Medical convention of Ohio, proceedings of, 429
Medical graduation, abolition of a rule in, 68
Medical resignation, 320
Medical topography of La Plata, 299
Medical topography of Central Arkansas, 321
Medical board of the army, 476
Medical and Surgical Journal in St. Louis, 476
Medical Journal in Havana, 476
Medical topography of Alabama, 473
Meningitis, 290
Menstrual fluid, analysis of, 59
Mercury, cyanuret of, in chancres, 385
Meteorology, 63
Meum and Teum, 317
Microscopic animalculæ, revivification of, 59
Milksickness, northern limits of, 65
Milksickness, true cause of, 73
Miscellaneous cases and observations, 332
Miscellaneous notices, 80
Mississippi river, bed and banks of, 237
Mississippi river, winter temperature of, 240, 314
Muriate of ammonia in scirrhus of the stomach, 463
Muscles, contusions of, 302
Muscles, syphilitic retraction of, 296

N

Naval hospital at Pensacola, 394
Navy ration, 397
Navy surgeons, 395
Navy yard at Pensacola, 394
New Orleans, climate of, 240
New process for anatomical injections, 295
New remedy for scalds and burns, 58
New work on southern diseases, 476
Nitrate of silver in bed sores, 386
Northern limits of milksickness, 65
Notices, miscellaneous, 80
Nursery treatment of infants, 293
Nutrition, abnormal, commonly called inflammation, 376

O

Objections to Liebig's theory of the uses of respiration and food, 51
Ohio medical convention, 315
Operation for enlarged patellar bursa, 229
Operation, Cæsarean, on a dwarf, 340
Os uteri, dropsy of, 392
Ovum, spermatozoa observed in, 465

P

Paine's materia medica, 237
Pallen on congestive fever, 412
Pamphleteers, anonymous, 319
Patellar bursa, operation for, 229
Patella, luxation of, 375
Percussion, 54
Pericarditis, Ballard's case of, 329
Phloridine, 52
Physicians of Dantzic sixty years since, 464
Pneumonia in the winter of 1841, 1842, 65
Pneumonia, the pure antimonial cure of, 368
Pneumonia produced by asphyxia, 422
Poisoning by snails, 357
Poisoning, case of, treated by electro-magnetism, 357
Population getting shorter, 306
Post-mortem researches, 241
Potassium, iodide of, in atonic ulcers, 299
Presumption of survivorship, 352, 442
Prevention of lead colic, 297
Primary syphilitic buboes, 459
Process for anatomical injections, 295
Proceedings of the medical convention of Ohio, 429
Professional correspondents, notice to, 79
Progress of temperance, 79
Proto-sulphuret of iron, new antidote for corrosive sublimate, 230
Pseudo-membranes, lymphatics in, 384
Public speaking, 312
Public speakers, throat-diseases of, 66
Pulmonary veins and air vesicles, communication of, 371
Pulmonary consumption in man and animals, 381

Q

Quadrupeds of North America, 318

Quinine in typhus, 386
Quinine in intermittent and remittent fevers, 390
Quinia, salts of, 286

R

Rage for invention, 468
Reform, temperance, assault on, 398
Remedy for scalds and burns, 58
Removal of Dr. Barton to Cuba, 313
Renal disease simulating vesical calculus, 467
Report on the New Orleans Charity Hospital, 159
Reports of the St. Louis Hospital, 402
Researches on digestion, 365
Researches, post-mortem, by Dowler, 241
Researches on the blood, 466
Retention of placenta, cathartics as a remedy for, 345
Retraction of muscles after syphilis, 296
Revivification of microscopic animalculæ, 59
Ricord on venereal diseases, 426
Robertson on caries of the teeth, 297

S

Salts of quinia, 286
Saturated solution of corrosive sublimate in erysipelas, 72
Sawyer's miscellaneous cases and observations, 332
Scalds and burns, new remedy for, 58
School for the blind, 77
Scirrhus of the stomach, muriate of ammonia in, 463
Sheep, effects of arsenic on, 462
Shoulder-joint, amputation of arm at, 405
Signs of maturity in new-born children, 61
Sleep of animals and man, 233
Snake-bites, 65
Southern diseases, new work on, 475
Spectacles, convex, in amaurosis, 457
Spermatozoa observed in the ovum, 465
Spinal marrow, tubercular disease of, 310
Steamboat bars, 238
Steamboat explosions, 240
Structure of the arteries, 458
Submarine thermometer, 285
Sulphite of the protoxide of platinum and sulphite of soda, a new double salt, 154
Surgeons in the Navy, 395
Surgical diagrams, 158
Survivorship, presumption of, 352, 442
Syphilitic retraction of muscles, 296
Syphilitic buboes, primary, 456
System of anatomy, by Wistar, 282

T

Tellkampf's case of amputation, 405
Temperance, progress of, 79
Temperance reform, assault on, 398
Temperature of the Mississippi in winter, 240, 314
Temperature and diseases of the year 1842, 64
Teeth, Robertson on caries of, 297
Tertiary formations, 238
Thermometer, submarine, 285
Therapeutical arrangement of the materia medica, 237
Throat disease of public speakers, 66
Tiger-Tail, death of, 315
Todd on cathartics in retained placenta, 345
Topography of Central Arkansas, 321
Topography of La Plata, 299
Travelling editorials, 235, 313, 393, 469
Treatment of uterine hemorrhages, tabular view of, 452
Treatment of hemorrhagic diathesis, 53
Treatment of infants in the nursery, 293
Treatment of itch at Berlin, 231
Treatment of nasal enlargement, 234
Treatment of pneumonia at Guy's Hospital, 368
Tubercular deposits in bronchial glands, 380
Tuberculous disease of spinal marrow, 310
Typhus, value of quinine in, 386

U

University of Alabama, 473
Use of cathartics in retained placenta, 345
Uterine hemorrhage, treatment of, 452
Uterus, engorgement of, 225
Uterus, dropsy of the mouth of, 392
Uterus, abscess in the walls of, 307

V

Vaccine lymph from the pustules produced by tartarized antimony, 466
Value of quinine in typhus, 386
Value of quinine in intermittent and remittent fevers, 390
Variety of false aneurism, 283
Veins, introduction of air into, 460
Venereal diseases, by Ricord, 426
Virey's objections to Liebig's theory of respiration, 51

W

Waste of hospital liquors, 398
West, diseases of, 236
Western meteorology, 63
Willoughby University of Lake Erie, 68
Winter and spring visits of invalids to New Orleans, 314
Winter temperature of the Mississippi, 240, 314
Wounds of the intestines, memoir on the nature and treatment of, 1, 81, 161

### RECEIPTS OF THE MEDICAL JOURNAL FOR MAY.

| | | |
|---|---|---|
| Dr. | W. H. Lee, Mexico, Mo. - - - - - - | $5 00 |
| | R. G. White, Pulaski, Tenn., - - - - - | 5 00 |
| | A. W. Webb, Columbia, Ark. - - - - - | 5 00 |
| | J. Morrison, Arcadia, Ill., - - - - - - | 5 00 |
| | J. P. Smith, Starkville, O., - - - - - | 5 00 |
| | —— Blackstone, Athens, O., - - - - - | 5 00 |

The WESTERN JOURNAL OF MEDICINE and SURGERY is published monthly by the undersigned, at the corner of Main and Fifth streets, Louisville, at $5 per annum, payable in advance. Each number contains from 80 to 84 pages making two volumes in the year of about 500 pages.

Contributions to its pages by the Physicians of the Valley of the Mississippi, addressed to the Editors, are respectfully solicited.

Letters on business, to be addressed, postage paid, to the publishers.

☞Postmasters, by the regulations of the Postoffice Department, will frank letters containing subscription money, and all remittances so franked are at the risk of the publishers.

*August*, 1843. PRENTICE & WEISSINGER.

